# Therapeutic Uses of Normal Saline

**Published:** 1902-08-16

**Authors:** J. A. Philip


### Hospital Clinics and Medical Progress.

### THERAPEUTIC USES OF NORMAL SALINE.

By J. A. Philip, M.D.

A method of treatment which merits further trial is the use of normal serum. It is, or should be, one of the essentials of an operating theatre, always ready for immediate use in cases of collapse, and its great value in such cases is undoubted, but I wish to say a little about its use in various septic or toxic conditions of the blood. As I have already written of its undoubted value in cases of puerperal fever, or in the milder forms of septicæmia, I shall here only point out that after an injection of normal serum, I have seen all the symptoms of a formidable attack of puerperal fever passing into peritonitis, disappear in a very few hours. This is well worth noting seeing how powerless we so often are when face to face with a case of puerperal fever of a severe type. There are other septic conditions such as erysipelas, poisoning from wounds, etc., in which, reasoning from analogy, normal serum is likely to be of use. When I used it first, it was merely to counteract the bad effects of blood-letting in debilitated subjects, but I quickly saw that it had powerful therapeutic properties, and then employed it alone. One would be hopeful of good results from its use in heat-stroke, as here we have probably, in addition to the heating of the blood, a retention of some organic toxins, which paralyze the vagus and other nerves. In three cases of severe heat-stroke, bleeding to from 8 to 12 ounces was resorted to, followed by the injection of 9 to 16 ounces of normal serum. In about three hours after, a considerable improvement had taken place, with passing of urine, perspiration, diarrhœa and vomiting. The pulse and breathing improved, convulsions became less frequent, cyanosis passed off, and the pupils became sensitive to light. In uræmic stupor with convulsions, normal serum is likely to re-establish the activity of the kidneys, and modify the toxic condition of the blood, especially if it be preceded by blood-letting; and puerperal eclampsia is another condition in which its use would seem to be indicated. Our means of influencing diabetic coma are so ineffective that one would gladly employ a remedy giving some hope of success, for hitherto the use of heroic doses of carbonate of soda is the only treatment attended by any success. I once treated an epileptic with large enemata of saline water—the only available remedy at the time—with the hope of cutting short the succession of fits to which he was at rare intervals liable. The attacks were due to syphilis, and were accompanied by severe renal pain and mental confusion in the intervals, warnings that the fits were not ended. The treatment was successful, as the general condition improved, and the fits did not return. My object in writing these brief notes is to induce others to continue experimenting with normal serum, particularly in puerperal fever, in which the results I have seen have been most encouraging, and to test its efficacy in other septic or toxic conditions of the blood, such as in erysipelas, carbuncle, pelvic cellulitis, or in cases of narcotic poisoning

